# Severe COVID-19 and non-COVID-19 severe sepsis converge transcriptionally after a week in the intensive care unit, indicating common disease mechanisms

**DOI:** 10.3389/fimmu.2023.1167917

**Published:** 2023-04-06

**Authors:** Andy Y. An, Arjun Baghela, Peter Zhang, Reza Falsafi, Amy H. Lee, Uriel Trahtemberg, Andrew J. Baker, Claudia C. dos Santos, Robert E. W. Hancock

**Affiliations:** ^1^ Centre for Microbial Diseases and Immunity Research, Department of Microbiology and Immunology, University of British Columbia, Vancouver, BC, Canada; ^2^ Department of Molecular Biology and Biochemistry, Simon Fraser University, Burnaby, BC, Canada; ^3^ The Department of Critical Care, Keenan Research Centre for Biomedical Science, St. Michael’s Hospital, University of Toronto, Toronto, ON, Canada; ^4^ Department of Critical Care, Galilee Medical Center, Nahariya, Israel

**Keywords:** COVID-19, sepsis, gene expression, immune dysfunction, longitudinal analyses

## Abstract

**Introduction:**

Severe COVID-19 and non-COVID-19 pulmonary sepsis share pathophysiological, immunological, and clinical features. To what extent they share mechanistically-based gene expression trajectories throughout hospitalization was unknown. Our objective was to compare gene expression trajectories between severe COVID-19 patients and contemporaneous non-COVID-19 severe sepsis patients in the intensive care unit (ICU).

**Methods:**

In this prospective single-center observational cohort study, whole blood was drawn from 20 COVID-19 patients and 22 non-COVID-19 adult sepsis patients at two timepoints: ICU admission and approximately a week later. RNA-Seq was performed on whole blood to identify differentially expressed genes and significantly enriched pathways.

**Results:**

At ICU admission, despite COVID-19 patients being almost clinically indistinguishable from non-COVID-19 sepsis patients, COVID-19 patients had 1,215 differentially expressed genes compared to non-COVID-19 sepsis patients. After one week in the ICU, the number of differentially expressed genes dropped to just 9 genes. This drop coincided with decreased expression of antiviral genes and relatively increased expression of heme metabolism genes over time in COVID-19 patients, eventually reaching expression levels seen in non-COVID-19 sepsis patients. Both groups also had similar underlying immune dysfunction, with upregulation of immune processes such as “Interleukin-1 signaling” and “Interleukin-6/JAK/STAT3 signaling” throughout disease compared to healthy controls.

**Discussion:**

Early on, COVID-19 patients had elevated antiviral responses and suppressed heme metabolism processes compared to non-COVID-19 severe sepsis patients, although both had similar underlying immune dysfunction. However, after one week in the ICU, these diseases became indistinguishable on a gene expression level. These findings highlight the importance of early antiviral treatment for COVID-19, the potential for heme-related therapeutics, and consideration of immunomodulatory therapies for both diseases to treat shared immune dysfunction.

## 1 Introduction

The COVID-19 pandemic has infected >650 million and killed 6-18 million people globally as of February 2023 (1, 2). While COVID-19 is caused by a novel virus (SARS-CoV-2), in its most severe form it has striking parallels to severe sepsis, a life-threatening organ dysfunction caused by a dysregulated host response to infection (3), which killed 11 million people in 2017 (4). While SARS-CoV-2 initially infects the lung, subsequent involvement of multiple organs accounts for most COVID-19 deaths (5). The concept of viral infections causing sepsis is not new (6) and culture-negative sepsis can be explained in part by the presence of underdiagnosed/underappreciated viral pathogens (7). Thus, many cases of severe COVID-19 are likely virus-associated sepsis.

This hypothesis is further supported by the observation that the hallmarks of sepsis immune dysfunction (overactive inflammatory response and enduring immunosuppression) (8) have been documented in severe COVID-19 (9), including increased cytokine expression (10) and T-cell deficits (11, 12). Furthermore, long-term outcomes of both sepsis (post-sepsis syndrome) and COVID-19 (“long COVID”) share multiple neurocognitive and immune deficits (13, 14). In addition, we recently showed that endotypes identified in a cohort of all-cause sepsis patients could also predict severity in COVID-19 patients, indicating further parallels in pathophysiology between the two diseases (15, 16). Compared to other viral diseases such as influenza, COVID-19 generally has higher TNFα/IL-1β-associated inflammation (17) and lower interferon responses (18).

Despite salient similarities, clear mechanistic and gene expression overlaps have not been demonstrated between severe sepsis and severe COVID-19, since most studies to date have lacked non-COVID-19 sepsis controls (19, 20). The rare studies that do include both groups of patients could show that certain COVID-19-associated features, such as autoantibody production, were related to sepsis severity and not unique to COVID-19 (21). Furthermore, since both sepsis and COVID-19 have been shown to be highly dynamic diseases involving immune perturbations (22, 23), comprehensive understanding requires analysis of more than a single timepoint to fully understand how these two diseases evolve.

If COVID-19 and sepsis caused by other pathogens were similar diseases, this could create therapeutic and prognostic opportunities and further support the application of decades of sepsis research into biomarkers, clinical care, and therapies to COVID-19 (as done during the pandemic). Likewise, new immunomodulatory therapies developed for COVID-19 may be repurposed and tested in sepsis patients, since sepsis currently has no specific treatment other than antibiotics and supportive care (24).

To determine whether severe COVID-19 was another form of severe sepsis at the mechanistic level, gene expression trajectories were compared between severe COVID-19 patients and otherwise clinically indistinguishable and contemporaneous non-COVID-19 severe sepsis patients in the ICU. While initial differences existed, particularly with regards to antiviral responses and heme metabolism, patients from both groups became virtually indistinguishable at the gene expression level after a week in the ICU, suggesting that these two diseases converge into the same pathophysiological processes that likely typify severe sepsis. These results further support labeling and treating severe COVID-19 as severe sepsis, particularly in the later stages of disease.

## 2 Materials and methods

### 2.1 Study design and sample collection

**Figure 1:**
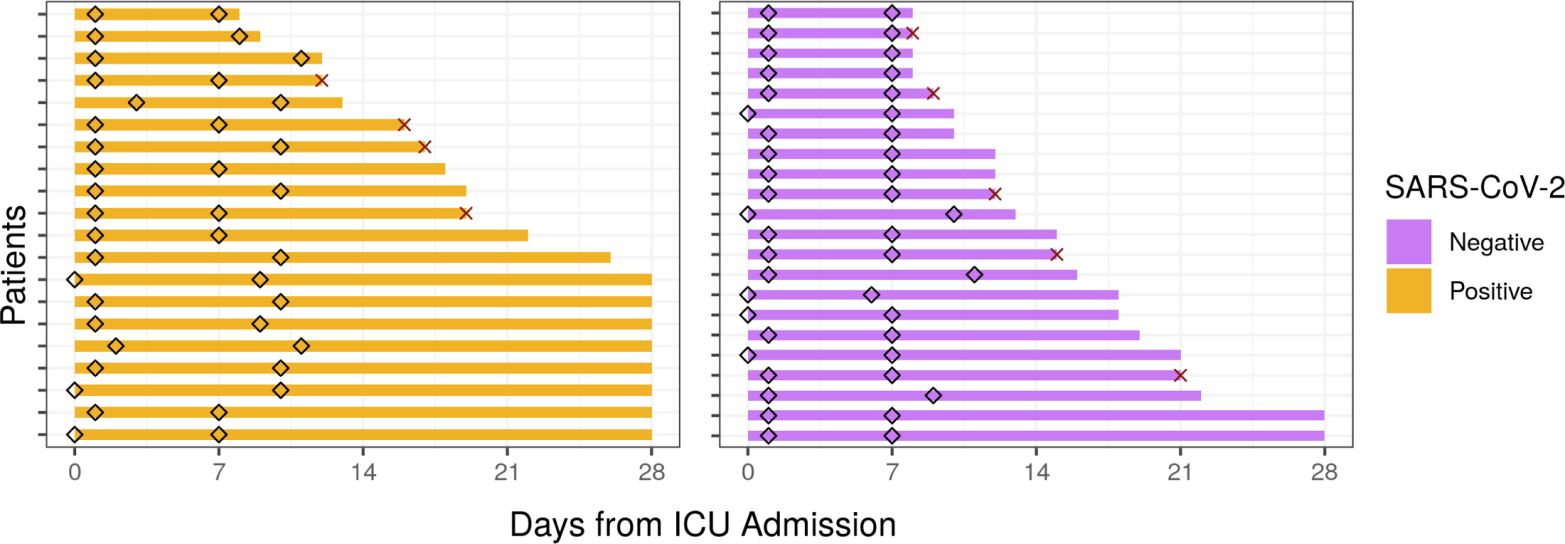
Sampling times and hospitalization duration of ICU patients from the COLOBILI cohort. Diamonds indicate time of sampling for each patient, with the majority of D1 samples collected at Day 1 in the ICU, and D7 samples at Day 7 in the ICU. The solid bars represent the duration of hospital stay (cutoff at 28 days post-ICU admission). X indicates death in the ICU.

As part of the prospective observational “COVID-19 Longitudinal Biomarkers of Lung Injury” (COLOBILI) study, 42 ICU adult (≥18 years) patients were consented and enrolled after ICU admission at St. Michael’s Hospital (Toronto, Canada) between March 2020 and February 2021 (**Table 1**). To be included in the analysis, patients satisfied three main inclusion criteria: 1) patients presented with respiratory deterioration from suspected COVID-19, 2) patients had a SOFA score ≥2 at ICU admission, and 3) patients had 2.5 mL of whole blood drawn into PaxGene Blood RNA tubes (BD Biosciences) at two timepoints, approximately Day 1 and Day 7 in the ICU (**Figure 1**; abbreviated D1 and D7). Exclusion criteria included: 1) age under 18 years old, 2) refusal to participate, 3) unknown 28-day mortality, 4) failure to obtain blood sample at admission, or 5) known to have had COVID-19 in the past 4 weeks. Samples were frozen and transported to Vancouver, Canada, for RNA isolation (PAXgene Blood RNA Kit; Qiagen) followed by RNA-Seq (**Supplemental Methods**). In addition, 5 healthy controls from Vancouver had blood collected and processed identically. After enrollment, 20 patients were determined to be SARS-CoV-2 positive based on PCR for SARS-CoV-2 RNA, while all 22 SARS-CoV-2 negative patients had at least two negative PCR tests (Supplemental Methods). SARS-CoV-2 positive patients had negative bacterial blood cultures at both timepoints. All patients satisfied Sepsis-3 criteria for sepsis (suspected/confirmed infection with a SOFA score ≥2 at ICU admission) (3). Nine patients (4 SARS-CoV-2 positive, 5 negative) died after their second blood draw but before 28 days in the ICU.

**Table 1:** Patient demographics of ICU patients in this study.

Clinical Variables	PCR Negative (22)	PCR Positive (20)	P-value
Patient Characteristics			
Age	56 ± 17.1 (22)	64 ± 11.8 (20)	0.140
Sex (Male)	72.7% (16/22)	80.0% (16/20)	0.723
28-Day ICU Survival (Yes)	77.3% (17/22)	80.0% (16/20)	1.000
Duration of ICU stay (Days)	18.6 ± 16.5 (22)	31.9 ± 24.3 (20)	**0.020**
Steroids During Hospitalization (Yes)	59.1% (13/22)	45.0% (9/20)	0.546
Body Mass Index	30.8 ± 12.5 (22)	28.2 ± 5.2 (20)	0.791
Illness Pre-Admission (Days)	8.2 ± 10.9 (18)	6.9 ± 5.5 (17)	0.230
Antibiotics Pre-Admission (Yes)	4.5% (1/22)	10.0% (2/20)	0.598
Smoker (Yes)	27.3% (6/22)	20.0% (4/20)	0.723
Race			0.073
African	9.1% (2/22)	10% (2/20)	
Asian	13.6% (3/22)	45% (9/20)	
European	9.1% (2/22)	0% (0/20)	
Latin, Central, South American	0% (0/22)	5% (1/20)	
North American Aboriginal	4.5% (1/22)	15% (3/20)	
Other North American	36.4% (8/22)	10% (2/20)	
Unknown	27.3% (6/22)	15% (3/20)	
Respiratory Comorbidities			
Asthma (Yes)	13.6% (3/22)	5.0% (1/20)	0.608
Obstructive Sleep Apnea (Yes)	13.6% (3/22)	15.0% (3/20)	1.000
Pneumonia (Yes)	4.5% (1/22)	15.0% (3/20)	0.333
COPD (Yes)	13.6% (3/22)	10.0% (2/20)	1.000
Bronchiectasis (Yes)	4.5% (1/22)	0.0% (0/20)	1.000
Previous Pulmonary Surgery (Yes)	9.1% (2/22)	0.0% (0/20)	0.489
Day 1 ICU Variables			
SOFA Score	9.7 ± 3.3 (22)	9.4 ± 3.2 (20)	0.849
Glasgow Coma Score	2.7 ± 1.6 (22)	3 ± 1.5 (20)	0.489
Respiratory SOFA Score component	2.4 ± 1 (22)	2.8 ± 0.6 (18)	0.312
PaO_2_/FiO_2_ Ratio	202.9 ± 101.1 (22)	171.2 ± 56.6 (18)	0.399
Admission APACHE II Score	26.4 ± 7.6 (22)	24.6 ± 8.2 (20)	0.278
On Mechanical Ventilation (Yes)	86.4% (19/22)	85.0% (17/20)	1.000
Given Antibiotics (Yes)	90.9% (20/22)	85.0% (17/20)	0.656
Blood Culture Positive (Yes)	22.7% (5/22)	0.0% (0/20)	**0.049**
White Blood Cells (10^3^ cells/µL)	11.6 ± 7.1 (22)	10.7 ± 5.3 (20)	0.980
Neutrophils (10^3^ cells/µL)	10 ± 6.6 (21)	8.9 ± 5.3 (20)	0.715
Lymphocytes (10^3^ cells/µL)	0.9 ± 0.8 (21)	0.9 ± 0.5 (20)	0.549
Monocytes (10^3^ cells/µL)	0.5 ± 0.3 (21)	0.5 ± 0.4 (20)	0.917
Eosinophils (10^3^ cells/µL)	0.1 ± 0.2 (21)	0.1 ± 0.1 (20)	0.910
Platelets (10^3^ platelets/µL)	156.1 ± 56 (22)	251.8 ± 124 (20)	**0.004**
Fibrinogen (g/L)	4.3 ± 2.3 (8)	3.9 ± 1.7 (4)	0.932
D-Dimer (ng/mL)	2969 ± 1875 (5)	2100 ± 1873 (4)	0.268
C-Reactive Protein (mg/L)	50.1 ± 41.3 (5)	150.8 ± 67.7 (7)	**0.035**
Lactate (mmol/L)	2.1 ± 1.3 (19)	1.8 ± 1.3 (18)	0.394
Day 7 ICU Variables			
SOFA Score	5.3 ± 3.4 (22)	8.3 ± 4.4 (20)	**0.026**
Glasgow Coma Score	2 ± 1.3 (22)	3 ± 1.2 (20)	**0.016**
Respiratory SOFA Score component	2.6 ± 0.5 (13)	2.7 ± 0.8 (17)	0.687
PaO_2_/FiO_2_ Ratio	197.3 ± 65.7 (13)	179.4 ± 72 (17)	0.439
On Mechanical Ventilation (Yes)	72.7% (16/22)	85.0% (17/20)	0.460
Given Antibiotics (Yes)	72.7% (16/22)	50.0% (10/20)	0.231
Blood Culture Positive (Yes)	0.0% (0/22)	0.0% (0/20)	1.000
White Blood Cells (10^3^ cells/µL)	10 ± 3.7 (21)	11.3 ± 3.8 (20)	0.341
Neutrophils (10^3^ cells/µL)	7.7 ± 3.4 (21)	8.8 ± 3.6 (20)	0.341
Lymphocytes (10^3^ cells/µL)	1.2 ± 0.7 (21)	1.2 ± 0.6 (20)	0.549
Monocytes (10^3^ cells/µL)	0.6 ± 0.3 (21)	0.8 ± 0.4 (20)	0.206
Eosinophils (10^3^ cells/µL)	0.1 ± 0.2 (21)	0.2 ± 0.2 (20)	0.347
Platelets (10^3^ platelets/µL)	210.4 ± 147.5 (21)	361.4 ± 182 (19)	**0.002**
Fibrinogen (g/L)	0.6 ± NA (1)	3.5 ± 2.6 (3)	0.371
D-Dimer (ng/mL)	NA	2061 ± 2908 (2)	NA
C-Reactive Protein (mg/L)	224.8 (1)	614.5 ± 272.2 (2)	0.540
Lactate (mmol/L)	1.5 ± 0.5 (7)	6 ± 12.1 (11)	1.000

SARS-CoV-2 PCR tests were administered to all patients. Those with a positive PCR test were classified as having severe COVID-19, while those with two negative PCR tests were classified as non-COVID-19 severe sepsis patients. For categorical variables, significance was tested using the Chi-squared test with Yates’s correction, or the exact Fisher test if any expected value was less than 5, and the percentage and fraction of patients fitting the category is displayed. For continuous variables, the Wilcoxon Rank-Sum test was used, and the mean ± standard deviation of the variable is displayed, with the number of patients assessed in brackets. Remdesivir was used in one patient and tocilizumab was used in two patients, all of whom were SARS-CoV-2 positive patients. For a subset of patients, fibrinogen, D-dimer, C-reactive protein, and lactate levels were measured within 24 hours of blood draw used for RNA-seq. Bolded P-values indicate significant differences (p <0.05). NA, not available. Some metadata was not available for all patients and statistics were calculated using only the samples with recorded values.

### 2.2 Bioinformatic and statistical analysis

The *DESeq2* package (25) was used to identify differentially expressed (DE) genes between different patient subgroups at D1 and D7. DE genes were defined as genes having an adjusted p-value <0.05 (Benjamini-Hochberg correction) and an absolute fold change ≥1.5. Sex and sequencing batch were included in the DESeq2 model to adjust for possible confounders. For trajectory (D7 vs. D1) comparisons, a paired differential expression analysis was performed, where patients were indexed to their previous sample, which controlled for individual underlying baseline differences (*e.g.*, genetics, comorbidities, etc.). DE genes were separated into up-/down-regulated genes and used for pathway enrichment with the Reactome database (26) and Molecular Signatures Database Hallmark gene sets (27). Significantly enriched pathways/gene sets after multiple comparison correction (Bonferroni and Benjamini-Hochberg, respectively) indicated key dysregulated biological processes. Further information can be found in the Supplemental Methods.

## 3 Results

### 3.1 Severe COVID-19 and non-COVID-19 severe sepsis patients were nearly clinically indistinguishable at ICU admission

Patients with or without COVID-19 did not significantly differ for age, sex, ethnicity, and mortality rate, as well as potential respiratory confounders such as smoking status, pre-existing respiratory disease, and rates of mechanical ventilation (**Table 1**). Indeed, COVID-19 patients were almost clinically indistinguishable (15/18 hospital parameters, such as SOFA and APACHE II scores for disease severity) from non-COVID-19 sepsis patients at ICU admission, except for significantly higher platelet counts (and, where assessed, C-reactive protein levels) and significantly lower blood culture positivity in COVID-19 patients. While mortality rates were similar between the two groups, COVID-19 patients appeared to have somewhat more severe disease progression, staying in the ICU for significantly longer and having significantly higher SOFA scores at D7. Since these samples were collected early in the pandemic (between March 2020 to February 2021), the lack of clinical knowledge on appropriate care for these patients may have factored into their more prolonged severe presentations. Furthermore, an ICU clinical cohort study found that sepsis patients with COVID-19 also had higher disease severity than those without (28). All patients had an initial SOFA score ≥2 and were admitted due to respiratory deterioration suspected to be caused by SARS-CoV-2, thus satisfying both the “organ failure” and “suspected or confirmed infection” criteria of the Sepsis-3 definition.

Viral respiratory infections predispose patients to secondary bacterial infections, although the incidence of co-infections in COVID-19 patients varies by study and population (29). Whether severe COVID-19 is viral-induced sepsis or becomes sepsis from a secondary infection is difficult to distinguish, since blood cultures, the gold standard for determining infection, can be negative for both bacterial and viral sepsis. Thus, we determined the possibility of bacterial co-infections in these COVID-19 patients. At D1, no COVID-19 (PCR positive) patients had a positive blood culture, while a significantly higher percentage (22.7%) of non-COVID-19 sepsis (PCR negative) patients were blood culture positive, despite similar levels of antibiotic use pre- and during hospitalization (**Table 1**). At D7, no patients had a positive blood culture, likely due to continued antibiotic use, although antibiotic use significantly decreased from 85% to 50% in COVID-19 patients (Chi-squared p-value = 0.02), compared to the non-significant decrease from 91% to 73% in non-COVID-19 sepsis patients (Chi-squared p-value = 0.12). Furthermore, nosocomial secondary infections in COVID-19 have been observed to develop around a week after admission (30), so the decrease in antibiotic use over time suggested that these patients likely had not yet developed a new nosocomial infection during hospitalization. Lastly, nosocomial infections were found to greatly increase mortality rates (30), yet similar 28-day mortality rates were observed between these two groups. These results suggest bacterial co-infection was unlikely/rare in this cohort of COVID-19 patients during sample collection, addressing this potential confounder.

Specific SARS-CoV-2 strain data was unavailable; however, based on sampling time and location (Toronto, Canada, between March 2020 to February 2021), it was likely that patients were infected with the ancestral strain, Alpha variant, or Beta variant. As well, because the samples were collected early in the pandemic, no patients were vaccinated and newer therapies such as remdesivir and tocilizumab were used in only three patients, all of whom were SARS-CoV-2 PCR positive. Corticosteroids were used in half of patients because patients were enrolled prior to corticosteroids becoming the standard of care for COVID-19; however, rates of use did not differ between COVID-19 and non-COVID-19 sepsis patients, addressing another potential confounder with regards to the dampening effects of corticosteroids on the immune response.

### 3.2 Severe COVID-19 and non-COVID-19 severe sepsis patients became transcriptionally indistinguishable after one week in the ICU

**Figure 2:**
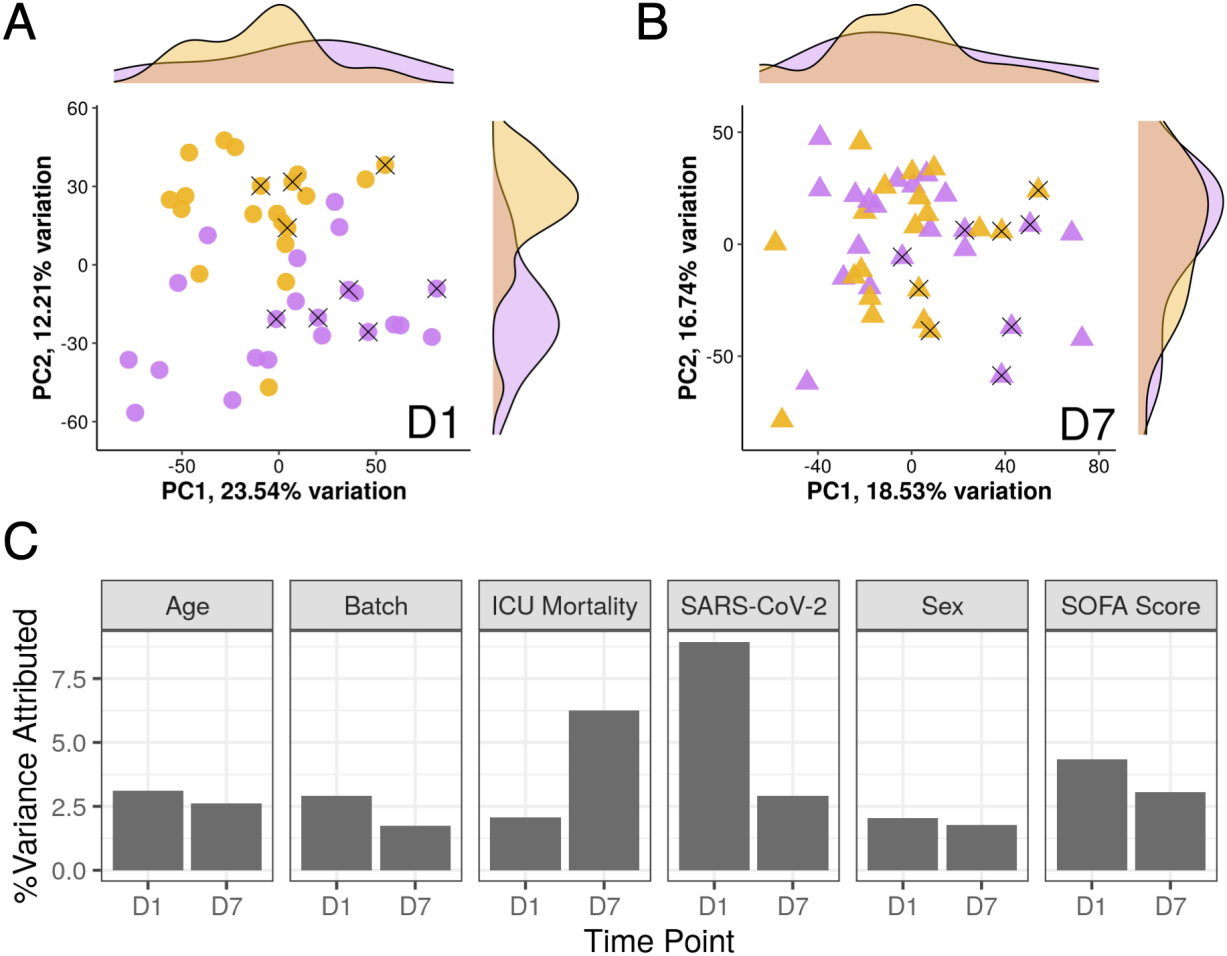
SARS-CoV-2 infection strongly influenced gene expression only at D1. Shown is a principal component analysis of analyzed ICU patients. The first two principal components were plotted and labelled based on COVID-19 status (yellow = SARS-CoV-2 positive, purple = SARS-CoV-2 negative) from D1 samples **(A)** and D7 samples **(B)**. X’s indicate patients who died in hospital. Density plots on the sides show the distribution of samples by COVID-19 status across the two principal components. **(C)** Percent variance of gene expression attributed to different metadata variables at D1 and D7. Notably, the percent variance attributed to SARS-COV-2 positivity strongly decreased from D1 to D7. In contrast, variance at D7 was more attributed to ICU mortality.

Since these patients initially appeared to be almost clinically indistinguishable, gene expression trajectories of COVID-19 and non-COVID-19 sepsis patients were then analyzed to determine if gene expression patterns were also similar. Principal component analysis (PCA) is an unsupervised clustering method that summarizes variation into principal components (PCs) representative of overall gene expression differences. PCA of ICU samples at D1 demonstrated that the percent gene expression variance attributed to whether a patient had COVID-19 or not was higher than other important metadata variables including age, sex, sequencing batch, SOFA score, and 28-day mortality (**Figure 2C**); this was reflected by the separation of COVID-19 and non-COVID-19 sepsis samples across PC2 (**Figure 2A**; see density plot to right). However, by D7, there was no obvious separation of the samples across PC1 or PC2 (**Figure 2B**), and the percent gene expression variance attributed to whether a patient had COVID-19 or not decreased substantially, while variance attributed to eventual 28-day mortality increased (**Figure 2C**).

**Figure 3:**
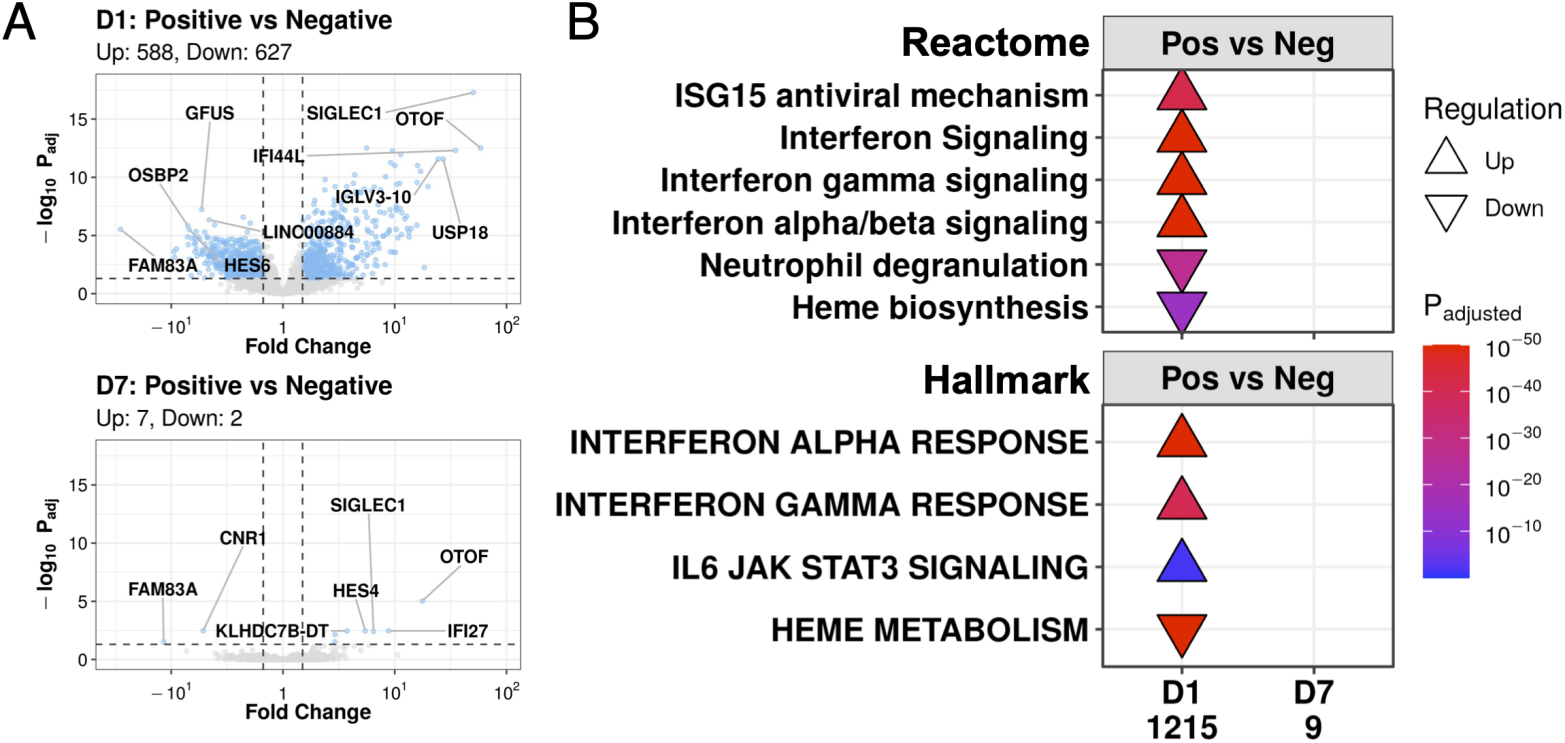
COVID-19 and non-COVID-19 sepsis patients differed at D1 but converged to nearly identical transcriptional profiles at D7. **(A)** Volcano plots of genes differentially expressed (DE) between COVID-19 (Positive) and non-COVID-19 sepsis (Negative) patients at D1 (top) and D7 (bottom). Coloured dots represent DE genes (absolute fold change ≥1.5, adjusted P-value <0.05; cut-offs indicated by dotted lines). The top 5 up- and down- regulated annotated genes (lowest adjusted p-value and highest fold change) are labelled. **(B)** Subset of enriched Reactome pathways (top) and Hallmark gene sets (bottom) using DE genes between COVID-19 (Pos) and non-COVID-19 sepsis (Neg) patients at D1 and D7, with all enriched pathways shown in **Figures S3**, **S4**. No pathways were enriched amongst the 9 DE genes at D7. “Upregulated” pathways/gene sets (Δ) had genes that were overrepresented in upregulated DE genes when compared to their prevalence in the genome, suggesting an increase in their function or activity, and vice versa for “downregulated” pathways/gene sets (∇). The total number of DE genes in each comparison are shown under each label.

Consistent with the PCA results, while COVID-19 and non-COVID-19 sepsis patients differed greatly by gene expression at D1 (1,215 DE genes), they became remarkably similar by D7 in the ICU, decreasing to only nine DE genes: *HES4*, *KLHDC7B*, *KLHDC7B-DT*, *OTOF*, *OR2B6*, *IFI27*, *SIGLEC1* (upregulated), *FAM83A*, and *CNR1* (downregulated) (**Figure 3A**). Since 28-day mortality was a source of gene expression variability at D7, the analysis was re-run with samples stratified by survival status to account for this potential confounder. Despite this stratification, at D7, there were still few DE genes detected in both non-survivors (1 DE gene, **Figure S1A**) and survivors (2 DE genes, **Figure S1B**) when comparing COVID-19 and non-COVID-19 sepsis patients. Thus, the contribution of COVID-19 status to gene expression changes decreased over time to the point where COVID-19 and non-COVID-19 patients became almost transcriptionally indistinguishable from each other after a week in the ICU.

### 3.3 Early antiviral response and suppressed heme metabolism processes distinguished severe COVID-19 from non-COVID-19 severe sepsis patients

Gene expression differences between COVID-19 and non-COVID-19 severe sepsis patients were evident at D1 in the ICU (**Figure 3A**). These DE genes at D1 enriched for viral response pathways from the Reactome database, including “ISG15 antiviral mechanism”, “Interferon signaling”, “Interferon-γ signaling”, and “Interferon α/β signaling”, which were all upregulated at D1 in COVID-19 patients vs. non-COVID-19 sepsis patients (**Figure 3B**). We also examined enrichment of Hallmark gene sets, which are sets of genes that represent specific well-defined biological states or processes and display coherent expression (27). These patterns were recapitulated for the “Interferon-γ response” and “Interferon-α response” gene sets, which showed identical enrichment patterns at D1 in severe COVID-19 patients (**Figure 3B**). In addition to differences in antiviral responses, COVID-19 patients, when compared to non-COVID-19 patients, also demonstrated downregulation at D1 of the “Heme biosynthesis” pathway and the “Heme metabolism” gene set (which includes genes involved in heme metabolism and erythroblast maturation-related processes) (**Figure 3B**). The nine DE genes at D7 between these two groups (**Figure 3A**) did not significantly enrich for any pathways or gene sets (**Figure 3B**).

**Figure 4:**
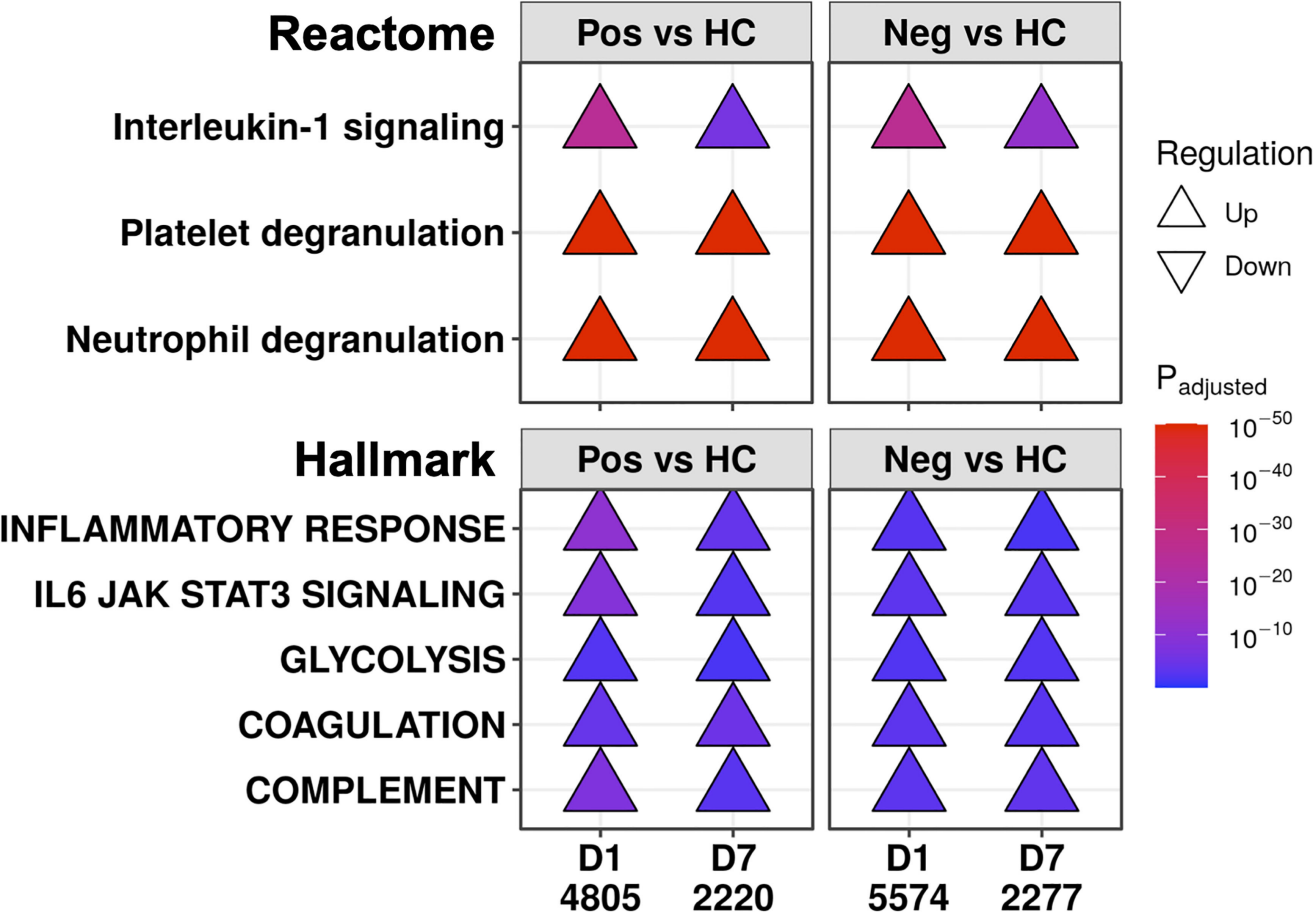
COVID-19 and non-COVID-19 sepsis patients shared immune and immune-related pathways at both D1 and D7. Subset of the enriched Reactome pathways (top) and Hallmark gene sets (bottom) using DE genes at D1 and D7 between COVID-19 (Pos) patients and healthy controls (HC), and between non-COVID-19 sepsis (Neg) patients and healthy controls. The full list of enriched pathways and gene sets are shown in **Figures S3**, **S4**. “Upregulated” pathways/gene sets (Δ) had genes that were overrepresented in upregulated DE genes when compared to their prevalence in the genome, suggesting an increase in their function or activity. The total numbers of DE genes in each comparison are shown under each label.

We then looked for underlying similar pathophysiology in these patients during the first week of the ICU by comparing COVID-19 and non-COVID-19 patients to healthy controls at each timepoint (selected examples in **Figure 4**; complete set in **Figures S3, S4**). Multiple immune pathways were upregulated at both timepoints in both COVID-19 and non-COVID-19 sepsis patients compared to healthy controls, including “Neutrophil degranulation” and “Interleukin-1 signaling”, as well as the gene sets “Inflammatory response”, “IL6-JAK-STAT3 signaling”, “Complement”, and “TNFα signaling *via* NF-κB”. For the “IL6-JAK-STAT3 signaling” and “Neutrophil degranulation”, there might have been additional differences in the magnitude of these processes early since these pathways were also relatively up- and down-regulated, respectively, at D1 in COVID-19 patients compared to non-COVID-19 sepsis patients (**Figure 3B**). “Glycolysis” (31, 32) and clotting processes such as the gene set “Coagulation” and the pathway “Platelet degranulation” (33, 34), can influence immune responses and have been strongly linked to sepsis and COVID-19. These were also upregulated at both timepoints in both COVID-19 and non-COVID-19 patients compared to healthy controls (**Figure 4**), highlighting further shared dysfunction.

**Figure 5:**
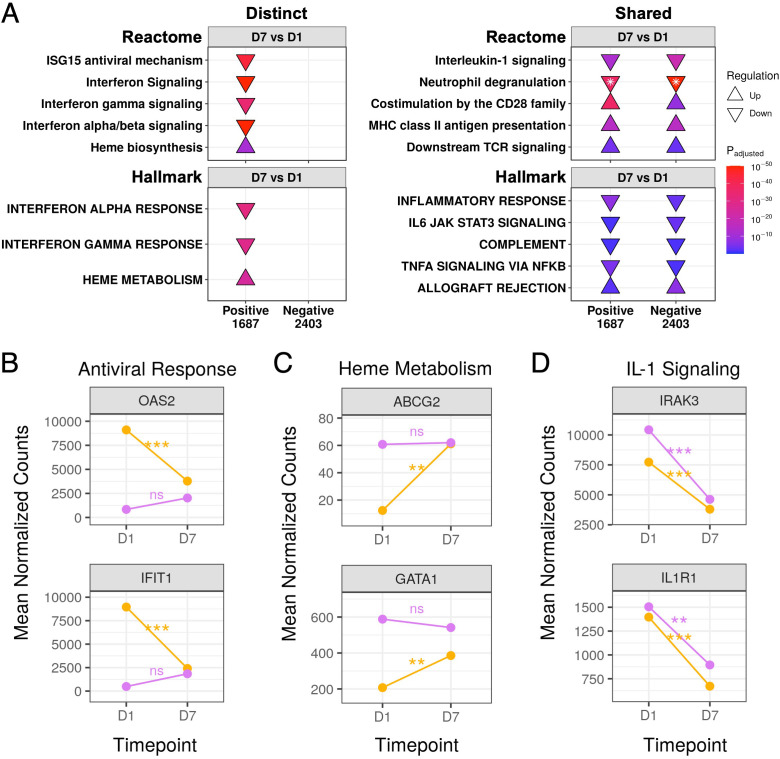
Gene expression trajectories of distinct and shared DE genes in COVID-19 and non-COVID-19 sepsis patients over time. **(A)** Subset of the enriched Reactome pathways (top) and Hallmark gene sets (bottom) using DE genes over time in COVID-19 (Positive) and non-COVID-19 sepsis (Negative) patients, separated into distinct (left) and shared (right) enriched pathways. The full list of enriched pathways/gene sets is shown in **Figures S4**, **S5**. “Upregulated” pathways/gene sets (Δ) had genes that were overrepresented in upregulated DE genes when compared to their prevalence in the genome, suggesting an increase in their function or activity, and vice versa for “downregulated” pathways/gene sets (∇). The total numbers of DE genes in each comparison are shown under each label. For one pathway, both up- and down- regulated genes were enriched (indicated by *); the direction with the lower adjusted p-value (more significantly enriched) is shown. The lower panels show mean DESeq2 normalized counts for representative genes involved in the antiviral response **(B)**, heme metabolism **(C)**, and interleukin-1 signaling **(D)**. Lines are coloured as yellow = SARS-CoV-2 positive, purple = SARS-CoV-2 negative. Genes in the antiviral response and heme metabolism significantly changed over time in COVID-19 patients but not in non-COVID-19 sepsis patients, while genes in interleukin-1 signaling significantly decreased over time in both patient groups. Statistically significant differences in panels B-D are indicated as ***p<0.001, **p<0.01, and *p<0.05; significance values were derived from DESeq2 model results. ns, not significant.

We also compared gene expression trajectories over time between COVID-19 and non-COVID-19 sepsis patients. Antiviral pathways and gene sets were downregulated over time only in COVID-19 patients (**Figure 5A**). This pointed to a robust anti-viral response only in COVID-19 patients at D1 which was substantially muted by D7, as represented by the temporal expression patterns of key antiviral and interferon-related genes such as *OAS2* and *IFIT1* (**Figure 5B**). These genes did not significantly change over time in non-COVID-19 sepsis patients and had low expression at both timepoints (**Figure 5B**).

Conversely, the “Heme biosynthesis” pathway and “Heme metabolism” gene set showed the opposite pattern, namely lower expression in COVID-19 patients compared to non-COVID-19 patients at D1 (**Figure 3B**) which significantly increased over time (**Figure 5A**). This was reflected by increased expression over time of genes involved in heme metabolism (such as *ABCG2* and *GATA1*) until they were no longer significantly differentially expressed compared to non-COVID-19 sepsis patients by D7 (**Figure 5C**). Non-COVID-19 sepsis patients conversely had elevated expression throughout disease that did not significantly change over time (**Figure 5C**).

There were also multiple shared inflammation-related pathways (“Neutrophil degranulation” and “Interleukin-1 signaling”) and gene sets (“Inflammatory response”, “IL6-JAK-STAT3 signaling”, “Complement”, and “TNFα signaling *via* NF-κB”) that decreased over time in both COVID-19 and non-COVID-19 sepsis patients (**Figure 5A**), as represented by shared decreases in gene expression in the IL-1 signaling genes *IRAK3* and *IL1R1* (**Figure 5D**). Analogously, both groups showed increases over time in adaptive immune pathways (“Co-stimulation by the CD28 family”, “MHC class II antigen presentation”, “Downstream TCR signaling”) and the “Allograft rejection” gene set (**Figure 5A**). These data, combined with multiple shared pathways when compared to healthy controls (**Figures 4**, **S3, S4**), suggested there were strong and similar overall background immune responses over time in both COVID-19 and non-COVID-19 sepsis patients.

Overall, two opposite gene expression trajectories were observed in COVID-19 patients but not in non-COVID-19 sepsis patients, representing an early antiviral response that decreased over time to levels seen in non-COVID-19 sepsis patients by D7, and an increase in heme metabolism that reached levels seen in non-COVID-19 sepsis patients by D7. Conversely, many immune and non-immune related pathways were shared between both groups and had similar trajectories (**Figures 4**, **5**, **S3–S5**). Thus, while COVID-19 and non-COVID-19 sepsis patients differed early during disease, their gene expression profiles became practically indistinguishable after ~1 week in the ICU, suggesting that by this stage, the disease process was extremely similar and dominated by severe sepsis mechanisms.

## 4 Discussion

The results from this study highlighted how severe COVID-19 and non-COVID-19 severe sepsis gene expression trajectories converge after an extended time in the ICU, which further supports existing symptomology and biomarker studies (35, 36) that severe COVID-19 is a form of viral-associated sepsis. Indeed, despite early gene expression differences, there was a strong underlying shared host response that was apparent at both D1 and D7 in both patient groups.

COVID-19 and non-COVID-19 sepsis patients initially differed at D1 with 1,215 DE genes but then became almost transcriptionally indistinguishable at D7 with only 9 DE genes (**Figure 3A**). Pathway enrichment using these DE genes identified biological processes related to these initial differences. At D1, COVID-19 patients had upregulation of antiviral signaling pathways and also downregulation of the “Neutrophil degranulation” pathway relative to non-COVID-19 sepsis patients (**Figure 3B**), suggesting a possible distinction between initial responses to the SARS-CoV-2 virus (interferons) and bacterial (neutrophil response) infections, consistent with proposed distinctions between early bacterial and viral sepsis signatures (37). These antiviral pathways decreased over time (**Figures 5A, B**), suggesting that the causative pathogen was likely no longer driving changes in leukocyte gene expression after a week in the ICU; this is consistent with results from a smaller COVID-19 cohort where the interferon response peaked early and decreased over time (38). This observation reiterates and reinforces the overall idea that severe sepsis is a dysregulated *host response* to infection, where the aberrant host response is the ultimate pathophysiological process that leads to symptoms, injury, and death, rather than the infection itself.

Since the pathogen-specific response appeared to peak early and wane over time, this likely explains why antiviral and monoclonal antibody therapies for COVID-19 are most effective early in the disease (39, 40), when a major driver of disease is the virus itself. For example, remdesivir given within the first 10 days after symptom onset led to a higher rate of recovery than when given 10 days after symptom onset (39) and is also effective when applied early in outpatients to prevent hospitalization (40). Monoclonal antibodies targeting the virus such as bamlanivimab, casirivimab, and imdevimab have also shown efficacy in outpatients at preventing hospitalizations (41), yet trials in hospitalized patients with severe COVID-19 (who are likely later in their disease progression) showed no difference when compared to standard of care (42). Thus, these findings provide biological evidence for the waning efficacy of these antivirals with time observed in clinical trials, which parallels the importance of early antibiotic use in bacterial sepsis, where each hour of antibiotic delay led to a mortality rate increase of 7.6% in septic shock (43). Further clinical trials for COVID-19 antivirals should focus on stratifying patients by disease stage or only testing new antiviral therapies on patients who were recently infected by COVID-19, since these antivirals are most likely to be effective under these circumstances.

In addition to antiviral pathways, heme metabolism appeared to be another differentiating factor among COVID-19 and non-COVID-19 sepsis patients. The “Heme metabolism” gene set and “Heme biosynthesis” pathways were highly expressed at both time points in non-COVID-19 sepsis patients (**Figure 5C**) and higher compared to COVID-19 patients at D1 (**Figure 3B**). Heme synthesis and hemoglobin assembly genes have also been documented to be upregulated in multiple sepsis datasets and are postulated to have cytoprotective functions in leukocytes (44). For example, heme metabolism in monocytes and macrophages is linked to a decreased inflammatory response and reduced oxidative stress (45). Thus, the high expression of heme metabolism observed in non-COVID-19 sepsis patients may be a compensatory mechanism for hyperinflammation, and this did not occur early on in COVID-19 patients.

The mechanism for this lack of enrichment of heme metabolism in COVID-19 patients early in disease is unclear. While heme metabolism is affected by hypoxia, mainly through reduced expression of heme oxygenase-1 and 2 (46), there was no significant difference in the PaO_2_/FiO_2_ ratio, which is a measurement of lung disease severity based on blood and lung oxygenation, between COVID-19 and non-COVID-19 sepsis patients (**Table 1**). Furthermore, the “Hypoxia” gene set was upregulated at both timepoints in all patients relative to healthy controls (**Figure S4**), suggesting the differential enrichment of heme metabolism pathways between COVID-19 and non-COVID-19 patients was not due to differences in hypoxia. It is, however, possible that the SARS-CoV-2 virus might directly interact with erythrocytes, hemoglobin, and heme, potentially disrupting heme metabolism (47). For example, the SARS-CoV-2 spike protein can bind to biliverdin, a metabolite of heme, to evade antibody responses (48). If interactions between SARS-CoV-2 and heme metabolism were occurring, this effect would be reduced later in the disease when the viral-related effects are no longer prominent, which could explain why the difference in heme metabolism disappears by D7 as COVID-19 patients elevate heme-related pathways to levels observed in non-COVID-19 patients (**Figures 5A, C**). Heme metabolism may be important clinically and therapeutically, since activating heme-oxygenase-1 through hemin suppressed SARS-CoV-2 replication *in vitro* (49), thus heme metabolism activation could potentially be another avenue for COVID-19 therapeutics.

While initial differences existed between severe COVID-19 and non-COVID-19 sepsis patients, there were multiple similarities in immune dysfunction throughout hospitalization. There was enrichment of “Neutrophil degranulation” and “Interleukin-1 signaling” pathways, as well as “Inflammatory response”, “Complement”, “TNFα signaling *via* NF-kB”, and “IL6-JAK-STAT3 signaling” gene sets (**Figure 4**) and other pathways (**Figures S3, S4**), by upregulated genes at both time points relative to healthy controls, suggesting an overall inflammatory milieu in both diseases during ICU hospitalization. These pathways decreased over time, coupled with an increase in adaptive immune functions (**Figure 5A**). Observing gene expression changes that indicated shared immune dysregulation, as well as other process that influence immunity, such as glycolysis and coagulation, supports observations from various clinical studies (31–34, 36, 50, 51) and highlights the possibility of applying immunomodulatory therapies that can treat both diseases.

For example, the “IL6-JAK-STAT3 signaling” gene set was upregulated in both groups at both timepoints when compared to healthy controls (**Figure 4**), but also relatively upregulated at D1 in COVID-19 patients compared to non-COVID-19 sepsis patients (**Figure 3B**). This was consistent with a proteomics study comparing differences in COVID-19 and bacterial acute respiratory distress syndrome (ARDS; often accompanies sepsis), which found that proteins involved in IL6-JAK-STAT3 signaling were elevated in COVID-19 (52). Tocilizumab, a monoclonal anti-IL6 receptor antibody (53), and baracitinib, a monoclonal antibody that inhibits JAK1 and JAK2 (which are activated in response to IL6 signaling) (54, 55), have also demonstrated initial clinical efficacy in COVID-19 patients. Since this pathway is also upregulated in non-COVID-19 sepsis patients compared to healthy controls, such treatments could be explored for all-cause sepsis, since they also demonstrate evidence of efficacy in *in vitro* and animal models (56, 57).

There are some limitations to our study. These results are from a single discovery cohort of unvaccinated, mostly male patients collected early in the pandemic, and should be validated by performing larger, sex-balanced studies that have both severe COVID-19 and concurrently collected non-COVID-19 severe sepsis patients. Vaccinated patients and those infected with current Omicron subvariants should be included in such a validation study to assess the impact of vaccination and new SARS-CoV-2 lineages. In addition, the sex imbalance in our study was unlikely to greatly affect detection of differences between COVID-19 and non-COVID-19 sepsis, because the male-to-female ratio was similar in both groups and we included sex as one of the covariates while detecting differentially expressed genes, accounting for this potential source of variation. Nevertheless, despite the modest sample size, thousands of DE genes were still identified, indicating the study was adequately powered for finding gene expression differences. Critically, these samples were paired, with two timepoints enabling indexing, that can help to eliminate various sources of patient heterogeneity that might otherwise overshadow true differential expression changes.

To conclude, severe COVID-19 is likely a form of viral sepsis since COVID-19 and non-COVID-19 severe sepsis patients had many commonly dysregulated genes just after ICU entry and became transcriptionally indistinguishable after a week in the ICU, which was only detectable by performing a longitudinal analysis. Antiviral pathways were elevated early in COVID-19 patients, highlighting the importance of early antiviral therapies for efficacy, while inhibition of IL-6 related mechanisms and other immunomodulatory therapies should be considered for both severe sepsis and severe COVID-19, since both diseases have similar underlying immune dysfunction, particularly later in disease. Heme metabolism activation might potentially be another novel avenue of COVID-19 treatment. Thus, these findings have clinical implications for the treatment of both COVID-19 and all-cause sepsis, as well as in potential future pandemics where severe sepsis is a common cause of death (58).

## Data availability statement

The datasets presented in this study can be found in online repositories. The names of the repository/repositories and accession number(s) can be found here: GSE185263, GSE222393 (GEO).

## Ethics statement

The studies involving human participants were reviewed and approved by Research Ethics Boards of St. Michael’s Hospital (REB#20-078) and University of British Columbia (REB#H20-02441). The patients/participants provided their written informed consent to participate in this study,

## Author contributions

RH, UT, AJB, and CS conceived the study. UT and CS contributed to the study design and were directly involved in sample and patient metadata collection in hospitals. AA and RH drafted the manuscript. AA, AB, and AL verified the quality and accuracy of sequencing data. AA performed bioinformatics analysis and wrote the initial draft of the paper. AA, AB, PZ, AL, and RH contributed to interpretation of data. RF processed samples for sequencing. RH was responsible for obtaining funding, led the study and extensively edited the paper. All authors contributed to the article and approved the submitted version.
