# Supplementary material for: Severe COVID-19 and non-COVID-19 severe sepsis converge transcriptionally after a week in the intensive care unit, indicating common disease mechanisms
Source: Front Immunol. 2023 Apr 6;14:1167917. doi: 10.3389/fimmu.2023.1167917 (PMC10115984; doi:10.3389/fimmu.2023.1167917)
Supplement: Supplementary file 1 [file DataSheet_1.docx]

Supplementary Material

Severe COVID-19 and non-COVID-19 severe sepsis converge transcriptionally after a week in the intensive care unit, indicating common disease mechanisms

Andy Y An, Arjun Baghela, Peter Zhang, Reza Falsafi, Amy H Lee, Uriel Trahtemberg, Andrew J Baker, Claudia C dos Santos, Robert EW Hancock

*** Correspondence:** Dr. Robert EW Hancock: bob@hancocklab.com

# Supplementary Methods

## Study design

The COVID-19 Longitudinal Biomarkers of Lung Injury study (COLOBILI) study (NCT04747782) was a prospective study created to follow patients from admission to the maximal severity phase and through convalescence. The study population was patients admitted to the critical care unit or general medicine ward at St. Michael's Hospital (Toronto, Canada), a tertiary referral medical center, with acute respiratory distress and suspected to have COVID-19.

Of the 142 enrolled patients, 42 ICU patients satisfied the three inclusion criteria to be part of this analysis: having two timepoints sequenced (sampled at approximately Day 1 and Day 7 in the ICU), having the second blood draw done prior to ICU discharge/death, and having a SOFA score ≥2 at ICU admission (**Figure S6**). Exclusion criteria included: age under 18 years old, refusal to participate, unknown 28-day mortality, failure to obtain the admission blood sample (*e.g.*, technical problems or identification of COVID-19 after ICU admission), or known to have had COVID-19 in the past 4 weeks. Immunosuppression was not an exclusion criterion; however, the use of corticosteroids did not differ between any of the comparisons (**Table 1**). These were not consecutively collected patients, as certain patients did not meet the previously listed criteria, usually due to having only one timepoint sequenced. Five healthy controls with no previous SARS-CoV-2 infection were also recruited from Vancouver, Canada, for baseline comparisons.

After enrollment in the study, all patients had a nasopharyngeal PCR performed (Altona RealStar SARS-CoV-2 RT-PCR Kit 1.0 or Cepheid GeneXpert Xpert Xpress SARS-CoV-2 assay), and intubated patients had an endotracheal aspirate performed as well (Seegene Allplex 2019-CoV Assay). All patients in the SARS-CoV-2 negative cohort had at least two negative tests performed to confirm their negativity. In this cohort, 20 patients were SARS-CoV-2 positive, while 22 patients were SARS-CoV-2 negative.

Informed consent was obtained from the patients or their legal representatives; in case that was not possible, the patients were enrolled using a deferred consent model and kept in the study until they regained capacity, or a surrogate decision maker was identified. This project was approved by the Research Ethics Boards of St. Michael’s Hospital (REB#20-078) and University of British Columbia (REB#H20-02441) and performed in accordance with the Helsinki Declaration of 1975.

## Blood collection and RNA-Seq

Approximately 2.5 mL of whole blood was drawn from each patient into PaxGene Blood RNA tubes (BD Biosciences, San Jose, CA, USA) at D1 and D7 in the ICU, for a total of 84 samples, collected between March 2020 to February 2021. Samples were frozen at -80°C and transported to Vancouver, Canada, for RNA isolation using the PAXgene Blood RNA Kit (Qiagen; Germantown, MD, USA) followed by sample preparation for RNA-Seq along with the healthy control samples. The Agilent 2100 Bioanalyzer (Agilent; Santa Clara, USA) was used for quantification and to check the quality of total RNA. The NEBNext Poly(A) mRNA Magnetic Isolation Module (NEB; Ipswich, USA) was used to capture poly-adenylated RNA, and cDNA libraries were prepared using the KAPA Total RNA HyperPrep Kit (Roche; Basel, Switzerland). RNA-Seq was performed on the Illumina Hi-Seq using single read runs of 150 base-pair long sequence reads (excluding adapter/index sequences).

## Differential gene expression analysis

All bioinformatic analyses were performed in the programming language R (v4.2.2) (1). A standard RNA-Seq processing pipeline was followed, including *FastQC* (v0.11.7) (2) and *MultiQC* (v1.6) (3) quality control, *STAR* (v2.6.0a) (4) alignment to the human genome (Ensembl GRCh38.92), and assessing read counts using *HTSeq-count* (v0.11.0) (5). All samples analyzed had more than one million total reads.

The count matrix was pre-filtered to remove globin genes (*HBA1*, *HBA2*, *HBB*, *HBD, HBG1, HBG2*) and low-count genes (mean counts across all samples <10) prior to differential expression analysis, resulting in a gene universe of 14,574 ENSEMBL gene IDs. The package *DESeq2* (v1.34.0) (6) was used to identify differentially expressed (DE) genes, which were defined as genes with an absolute fold change ≥1.5 and adjusted p-value <0.05 (Benjamini-Hochberg multiple test correction). Sequencing batch and sex were modelled as covariates in the *DESeq2* model, and the Wald test was used for hypothesis testing. Other covariates (*e.g.*, respiratory comorbidities, smoking, race) were not added as covariates due to the lack of statistical difference of these covariates between COVID-19 and non-COVID-19 patients, complexity to model due to multiple variables, and to reduce overfitting of the model from too many added covariates. For D7 vs. D1 comparisons, a paired differential expression analysis was performed. This was done by investigating the effects of conditions in the particular groups, with individuals nested within the groups (as outlined in the DESeq2 vignette) (6); the group was SARS-CoV-2 positivity, and condition was the timepoint. Essentially, patients were indexed to their previous sample, which controlled for individual underlying baseline differences (e.g., genetics, comorbidities, etc.).

To identify how patients clustered based on gene expression patterns, principal component analysis (PCA) was performed. PCA is an unsupervised clustering approach that decomposes multivariate data into multiple representative orthogonal principal components (PCs), which are numbered based on the proportion of variation they explain (*i.e.*, PC1 explains the largest variation, PC2 explains the second largest, etc.). To generate PCA plots and correlation plots, the *PCAtools* package (v2.6.0) (7) was used. The bottom 10% of genes with low variance was removed prior to PCA to focus on genes that vary due to condition, timing, and other metadata variables. Percent variance of gene expression attributed by metadata variables of interest were calculated by using the package *variancePartition* (v1.28.3) to calculate the proportion of variance attributed by each variable to each principal component (which uses a mixed linear model), which was then summed across all the principal components, weighted by the amount of variance explained by each principal component.

## Pathway enrichment analysis

The Reactome database (8) is an open-source, peer-reviewed pathway database of genes and proteins categorized into biological pathways hierarchies (e.g., “Immune System” > “Cytokine Signaling” > “Signaling by Interleukins” > “IL-1 Family Signaling” > Genes such as *IL1R1*, *IL1R2*, *ILRN*, etc.). To enable enrichment of more specific and biologically relevant Reactome pathways, DE genes were analyzed using the *SIGORA* package (v3.1.1) (9), which decreases the chance of observing multiple similar and overlapping pathways by analyzing gene pairs rather than individual genes (which may be present in overlapping pathways). Reactome pathways were considered significantly enriched with an adjusted p-value <0.001 (Bonferroni multiple test correction) as recommended in *SIGORA*.

To supplement and validate *SIGORA* pathway enrichment results, another database using a different enrichment method was performed. The Molecular Signatures Database (MSigDB) contains Hallmark gene sets, which are gene sets that represent “specific, well-defined biological states or processes with coherent expression” (10); these included gene sets such as “Inflammatory Response” or “Angiogenesis”. Gene sets were considered significantly enriched with an adjusted p-value <0.05 (Benjamini-Hochberg multiple test correction) and q-value <0.2, based on the default settings of the *enricher* function in the package *clusterProfiler* (v4.2.2) (11), with the gene universe as filtered genes in the count matrix.

Enrichment was performed separately on up- and down-regulated DE genes. Pathways and gene sets were considered “upregulated” if the genes in these pathways or gene sets were overrepresented in upregulated DE genes when compared to their prevalence in the gene universe, suggesting an increase in their function or activity, and vice versa for “downregulated”.

# Supplementary References

1. R Core Team. R: A language and environment for statistical computing [Internet]. Vienna, Austria: R Foundation for Statistical Computing; 2022 [cited 2022 Oct 18]. Available from: https://www.r-project.org/

2. Babraham Bioinformatics - FastQC A Quality Control tool for High Throughput Sequence Data [Internet]. [cited 2022 Jan 29]. Available from: https://www.bioinformatics.babraham.ac.uk/projects/fastqc/

3. Ewels P, Magnusson M, Lundin S, Käller M. MultiQC: summarize analysis results for multiple tools and samples in a single report. *Bioinformatics*. 2016 Oct 1;32(19):3047–8.

4. Dobin A, Davis CA, Schlesinger F, Drenkow J, Zaleski C, Jha S, et al. STAR: ultrafast universal RNA-seq aligner. *Bioinformatics*. 2013 Jan 1;29(1):15–21.

5. Anders S, Pyl PT, Huber W. HTSeq—a Python framework to work with high-throughput sequencing data. *Bioinformatics*. 2015 Jan 15;31(2):166–9.

6. Love MI, Huber W, Anders S. Moderated estimation of fold change and dispersion for RNA-seq data with DESeq2. *Genome Biol*. 2014 Dec 5;15(12):550.

7. Blighe K, Lun A. PCAtools: everything Principal Component Analysis [Internet]. 2022 [cited 2022 Oct 18]. Available from: https://github.com/kevinblighe/PCAtools

8. Fabregat A, Sidiropoulos K, Viteri G, Forner O, Marin-Garcia P, Arnau V, et al. Reactome pathway analysis: a high-performance in-memory approach. *BMC Bioinform*. 2017 Mar 2;18(1):142.

9. Foroushani ABK, Brinkman FSL, Lynn DJ. Pathway-GPS and SIGORA: identifying relevant pathways based on the over-representation of their gene-pair signatures. *PeerJ*. 2013 Dec 19;1:e229.

10. Liberzon A, Birger C, Thorvaldsdóttir H, Ghandi M, Mesirov JP, Tamayo P. The Molecular Signatures Database (MSigDB) hallmark gene set collection. *Cell Syst*. 2015 Dec 23;1(6):417–25.

11. Yu G, Wang LG, He QY. clusterProfiler: an R package for comparing biological themes among gene clusters. *OMICS*. 2012 May 3;16(5):284–7.

# Supplementary Figures and Tables

## Supplementary Figures

##
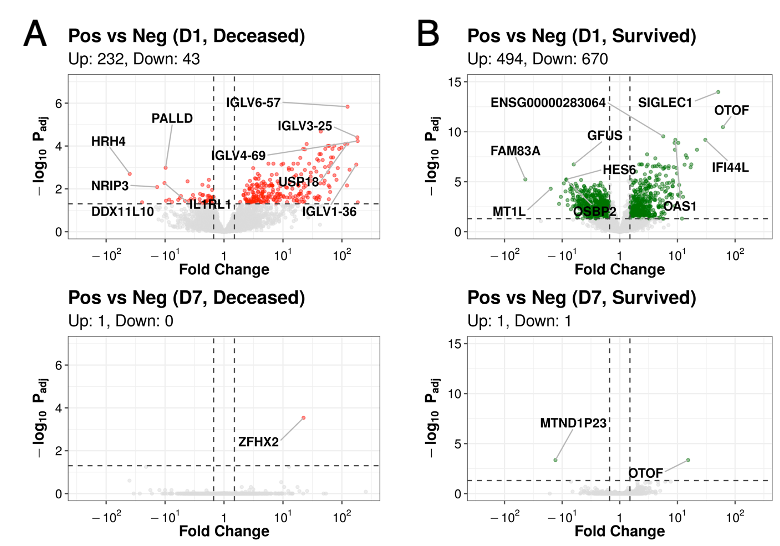


Supplementary Figure 1. Differential gene expression, separated based on eventual mortality, in ICU patients with and without COVID-19 at each timepoint, still showed few differences at D7. Volcano plots show

A: Comparison between COVID-19 and non-COVID-19 sepsis non-survivors at D1 and D7. B: Comparison between COVID-19 and non-COVID-19 sepsis survivors at D1 and D7. Coloured dots represent DE genes (Red = Deceased, Green = Surviving; absolute fold change ≥1.5, adjusted P-value <0.05; cut-offs indicated by dotted lines). The top 5 up- and down- regulated genes (lowest adjusted p-value and highest fold change) are labelled.

**
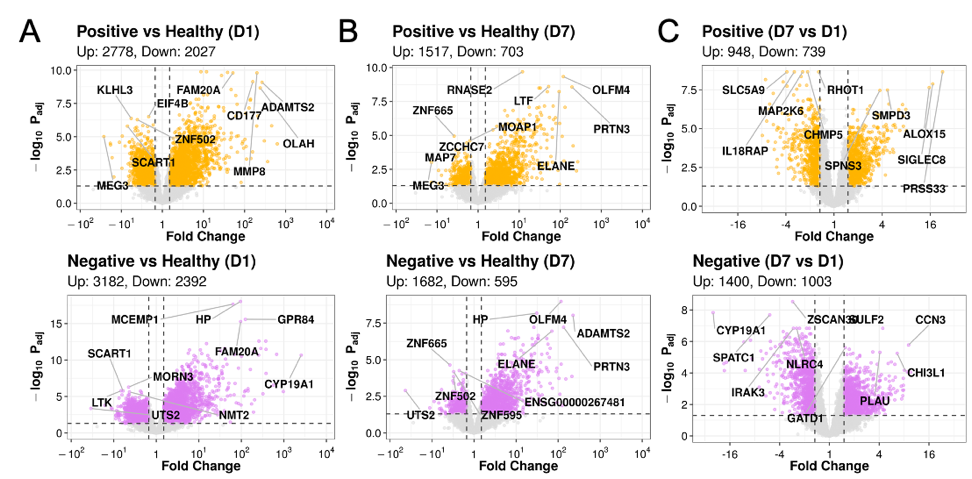
**

Supplementary Figure 2. DE genes in ICU patients with and without COVID-19 compared to healthy controls and over time. Volcano plots show

**A:** Comparison at D1 between COVID-19 (Positive; yellow dots) to healthy controls (top) and non-COVID-19 sepsis (Negative; purple dots) patients to healthy controls (bottom). **B:** Comparison at D7 between patients and healthy controls. **C:** Comparison between COVID-19 (top) and non-COVID-19 sepsis (bottom) patients over time. Coloured dots represent DE genes (absolute fold change ≥1.5, adjusted p-value <0.05; cut-offs indicated by dotted lines). The top 5 up- and down- regulated genes (lowest adjusted p-value and highest absolute fold change) are labelled.


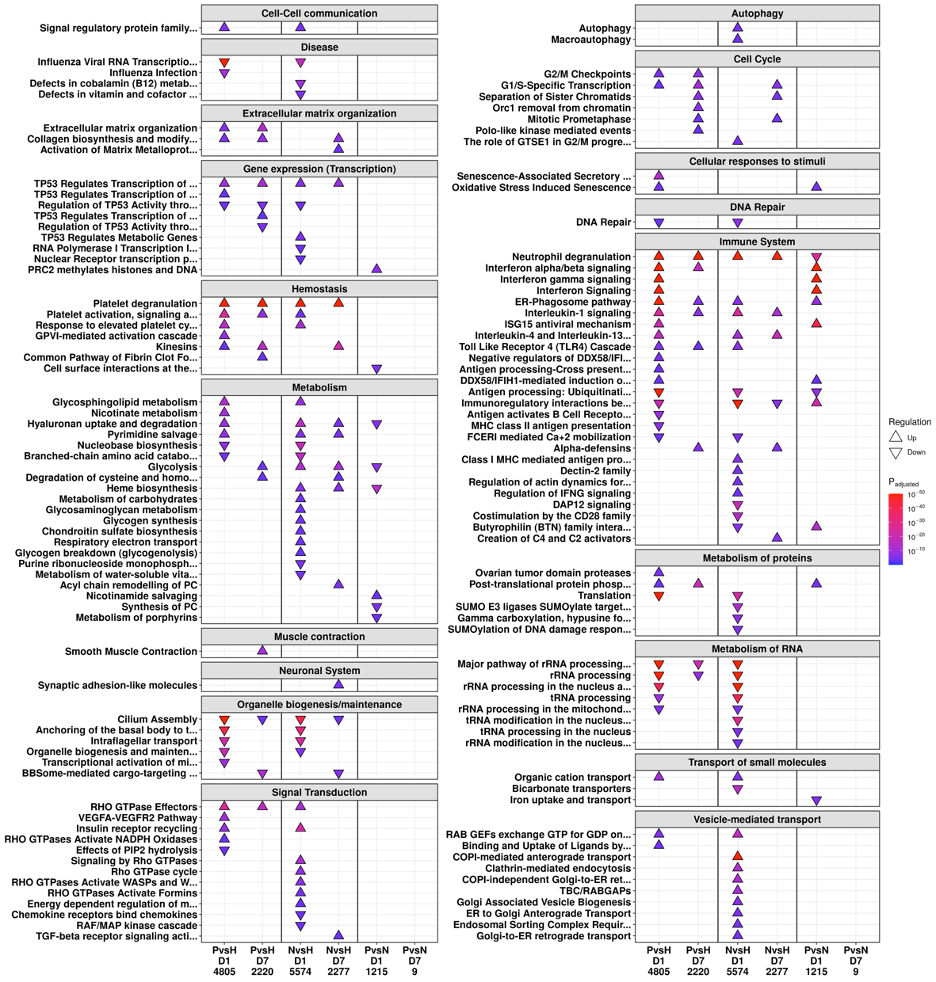


**Supplementary Figure 3.** **All enriched REACTOME pathways from DE genes of COVID-19 and non-COVID-19 sepsis patients, compared to healthy controls and each other at D1 and D7.** A subset of these pathways is shown in **Figures 3B** and **4**. P: SARS-CoV-2 positive, N: SARS-CoV-2 negative, H: healthy controls. Pathways were considered upregulated (Δ) if the genes in this pathway were overrepresented in upregulated DE genes when compared to their prevalence in the genome, suggesting an increase in their function or activity, and vice versa for downregulated (∇). P-values were adjusted for multiple comparisons using the Bonferroni correction with an adjusted p-value cut-off <0.001. The total number of DE genes in each comparison are shown under each label.


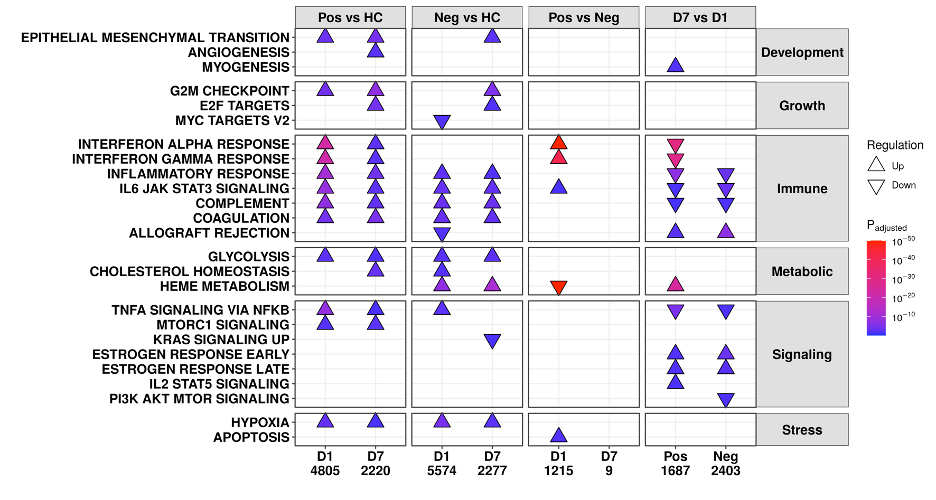


**Supplementary Figure 4.** **All enriched Hallmark gene sets from DE genes of COVID-19 and non-COVID-19 sepsis patients, compared to healthy controls and each other at D1 and D7, and over time.** A subset of these pathways is shown in **Figures 3B**, **4,** and **5A**. Pos: SARS-CoV-2 positive, Neg: SARS-CoV-2 negative, HC: healthy controls. Gene sets were considered upregulated (Δ) if the genes in this gene set were overrepresented in upregulated DE genes when compared to their prevalence in the genome, suggesting an increase in their function or activity, and vice versa for downregulated (∇). P-values were adjusted for multiple comparisons using the Benjamini-Hochberg correction, with an adjusted p-value cut-off <0.05. The total number of DE genes in each comparison are shown under each label.


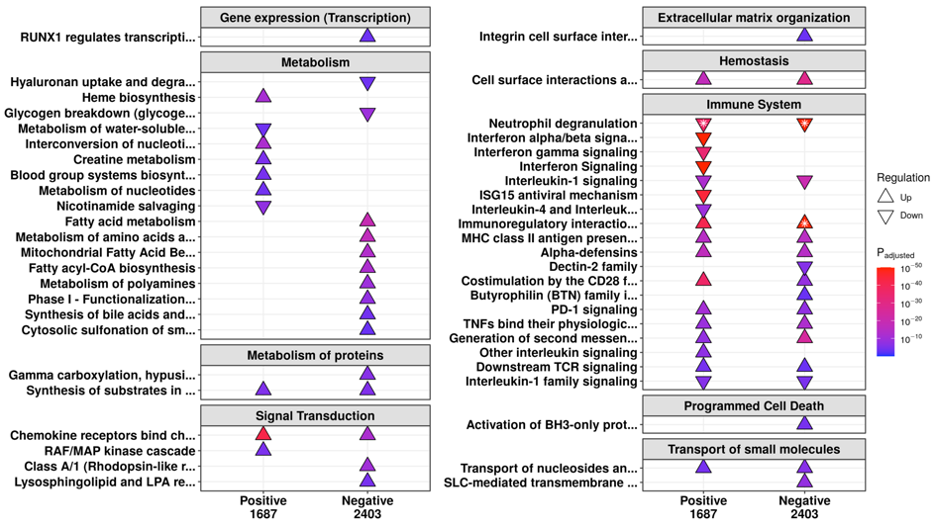


**Supplementary Figure 5.** **All enriched Reactome pathways from DE genes over time in COVID-19 and non-COVID-19 sepsis patients.** A subset of these pathways is shown in **Figure 5A**. Pathways were considered “upregulated” if the genes in this pathway were overrepresented in upregulated (Δ) DE genes when compared to their prevalence in the genome, suggesting an increase in their function or activity, and vice versa for downregulated (∇). P-values were adjusted for multiple comparisons using the Bonferroni correction with an adjusted p-value cut-off <0.001. For certain pathways, both directions were enriched (indicated by *); the direction with the lower adjusted p-value (more significantly enriched) is shown. The total number of DE genes in each comparison are shown under each label.

**Supplementary Figure 6.** **Flowchart of sample selection for analysis.** 142 hospitalized patients were enrolled in the COLOBILI study at St. Michael's Hospital (Toronto, Canada), a tertiary referral medical center, with acute respiratory distress and suspected to have COVID-19. Of these patients, 100 were not analyzed further, with the main reason being the lack of a second sample collected between 6-11 days after ICU admission. All patients had a nasopharyngeal PCR performed, and intubated patients had an endotracheal aspirate PCR performed as well. All patients in the SARS-CoV-2 negative cohort had at least two negative tests performed to confirm their negativity. In addition to these ICU patients, five healthy controls were also sequenced together and analyzed.
